# Socio-demographic and mental health correlates of internet addiction amongst Hong Kong university students under COVID-19

**DOI:** 10.3389/fpsyg.2023.1248378

**Published:** 2023-08-21

**Authors:** Daniel T. L. Shek, Wenyu Chai, Diya Dou, Lindan Tan, Tingyin Wong, Kaiji Zhou

**Affiliations:** Department of Applied Social Sciences, The Hong Kong Polytechnic University, Kowloon, Hong Kong SAR, China

**Keywords:** internet addiction, socio-demographic correlates, psychological morbidity, university students, COVID-19 pandemic, Hong Kong

## Abstract

**Introduction:**

Regarding the problem of Internet addiction (IA) amongst university students under the pandemic, there are several research gaps. Firstly, few studies have examined IA of university students in Hong Kong, which is a Chinese society heavily influenced by Western values. In addition, findings on the socio-demographic correlates and psychological well-being predictors of IA in university students are unclear. Finally, researchers have not systematically examined the interaction effects of socio-demographic factors (particularly gender and personal infection of COVID-19) and psychological morbidity on IA. This pioneer study aimed to investigate the predictive role of socio-demographic factors and psychological morbidity in IA, and the moderating effects of gender and personal infection of COVID-19 on the relationship between psychological morbidity and IA.

**Methods:**

We conducted an online survey (*N* = 1,020 university students) during the ending phase of Wave 5 of the pandemic in Hong Kong (late 2022 to early 2023). Socio-demographic correlates included age, gender, living status, personal and family financial situation, student status, personal and family infection of COVID-19. Participants responded to validated measures of psychological morbidity, including depression, suicidal behavior, and hopelessness. Hierarchical regression and simple slope analyses were used to examine the predictive role of socio-demographic variables and psychological morbidity in IA and the interactive effect of gender and personal infection of COVID-19 with psychological morbidity on IA.

**Results:**

Personal financial difficulty was a significant socio-demographic predictor of IA. Depression, suicidal behavior, and hopelessness positively predicted IA. We also found a significant interaction effect of gender and psychological morbidity on IA. While the predictive relationship between depression and IA was stronger in males than in females, hopelessness was more strongly related to IA in females than in males. Finally, there was a significant interaction effect of personal infection of COVID-19 and suicidal behavior on IA.

**Conclusion:**

Personal financial difficulty was a socio-economic correlate of IA. Psychological morbidity also predicted IA. Gender and personal infection of COVID-19 moderated the linkage between psychological morbidity and IA. The findings of the study enhance our understanding of individual differences in IA in university students during the pandemic, particularly concerning different ecological risk factors.

## 1. Introduction

During COVID-19, the Internet becomes the primary source of information, entertainment, and communication. Since March 2020, nearly all modes of entertainment, social services, academic learning, and career have transitioned online (Shek, 2021). As a result, many people, particularly university students, have become increasingly dependent on Internet usage. Some students may engage in problematic and excessive use of the Internet as a coping mechanism for emotional stress from the pandemic (Tomczyk and Lizde, 2023), which may predispose them to develop Internet addiction (IA). IA refers to excessive involvement in using the Internet, leading to maladaptive behavior such as impairment of psychosocial functioning (Shek et al., 2023a, 2023b).

However, not all students using the Internet develop IA problems. There are individual differences in IA, potentially due to risk factors (i.e., factors that increase the chance of IA such as negative mental health and other developmental disorders; Sulla et al., 2023; Zhao et al., 2023), and protective factors (i.e., factors that reduce the chance of IA occurrence such as resilience; Shek et al., 2023b) in different ecological systems. In this paper, we examined socio-demographic and mental health correlates of IA in university students in Hong Kong. Although there are negative impacts of the pandemic on young people, there are few studies on university students in Hong Kong (Shek, 2021; Shek et al., 2022a, 2022b). As such, the present study examined the socio-demographic correlates (gender, age, living status, international versus local student, economic challenges, and infection of COVID-19) and psychological morbidity (depression, hopelessness, and suicidal behavior) correlates of IA in university students in Hong Kong. Identifying socio-demographic and psychological morbidity correlates of IA is important for devising appropriate prevention and intervention programs for vulnerable groups during the pandemic (Shek, 2021; Shek et al., 2023c).

### 1.1. Socio-demographic factors related to IA

Researchers have identified some socio-demographic correlates of IA. First, the relationship between gender and IA among students during the COVID-19 pandemic is complex. Gavurova et al. (2022) revealed significant gender difference in IA among Slovak college students (*N* = 1,677), with males showing higher levels of overall IA and its subdomains including neglect of work and social life, and lack of control. Similarly, compared to female secondary school students in Nigeria, males showed a roughly 2-fold higher likelihood of being Internet addicted (N = 851; Onukwuli et al., 2023). These findings are consistent with research prior to the pandemic indicating higher rates of IA among male students (Liang et al., 2016; Vigna-Taglianti et al., 2017). However, Mengistu et al. (2023) revealed an opposite trend by showing that only females were positively associated with problematic smartphone use during the pandemic. Besides, Shek et al. (2023a) showed that gender was not related to IA in university students during the pandemic. In short, research findings on the relationship between gender and IA during the pandemic remain unclear.

Second, research showed that age is related to IA, with younger college students being more susceptible to IA than older students during COVID-19 (Gavurova et al., 2022). Malůš and Ciencialová (2021) found that age was negatively related to both smartphone addiction and IA under COVID-19 in 988 female undergraduate students. Shek et al. (2023b) also reported that younger students showed a higher level of IA than did older students. The “Strength and Vulnerability Integration” (SAVI) model (Charles, 2010) suggested that older individuals use regulation skills like “attentional strategies, appraisals, and behaviors” (p. 2) to manage daily emotional experiences and mitigate adverse events, which can reduce their risk of IA. However, some studies have shown no association between age and IA (Chi et al., 2020; Olawade et al., 2020).

Third, studies have shown that international students exhibit more psychological problems (Chen et al., 2020; Ahorsu et al., 2021; Maleku et al., 2021) and higher IA (Kaur and Chowdhury, 2023) during the pandemic than local students, likely due to the additional challenges they face, such as cultural adjustment (Kaur and Chowdhury, 2023). However, in Hong Kong, local students were found to be more stressed, anxious, and depressed (Shek et al., 2022a, 2022b) than international students during the pandemic, possibly due to the psychological trauma of the “social event” in 2019 (Shek, 2020).

Fourth, studies showed that living alone (Achab et al., 2011; Chi et al., 2020; Savolainen et al., 2020) is a risk factor for IA during the pandemic. For example, a study on 1,477 youth in Vietnam showed that living alone was associated with a higher risk of IA (Nguyen et al., 2023). In addition, compared to those living with family or roommates, Hong Kong university students who lived alone experienced more severe stress, anxiety, and depression during the epidemic (Shek et al., 2022a), which might trigger their higher IA. This observation can probably be explained by the social support hypothesis, which suggests that living with others can provide social support that can help young people to cope with the stress of the pandemic, reducing psychological problems and problem behavior such as IA.

Fifth, studies also showed that economic challenges are related to IA. Before COVID-19, studies on the relationship between family income and IA showed inconsistent findings: while some studies have shown a positive relationship (Ak et al., 2013; Lai and Kwan, 2017), others have shown a negative relationship (Islam and Hossin, 2016; Faltýnková et al., 2020) or a U-shaped relationship (Ahmadi, 2014). Studies conducted during the pandemic seem to find a general trend indicating a negative relationship between family income and adolescents’ IA or problematic computer use (Ripon et al., 2022; Sayeed et al., 2023). Additionally, financial loss and difficulty were also positively correlated with IA during the pandemic (Shek et al., 2023a).

Finally, infections of the COVID-19 in adolescents and their family members were also related to IA. Oka et al. (2021) found that being infected with the COVID-19 virus increased an individual’s risk (5.67 times greater risk) of developing Internet gaming disorder, probably because of greater stress and using the Internet as a coping. Besides, studies have shown that family members’ COVID-19 infection or loss of family members because of COVID-19 (Ripon et al., 2022) had a significant association with an individual’s addictive behaviors (Kamaşak et al., 2022). As infection in oneself or family members can be regarded as a traumatic event, it can be argued that infection would lead to addictive behavior such as IA among adolescents who had dysfunctional coping abilities (Brasso et al., 2022).

### 1.2. Psychological morbidity and IA

There are studies showing that psychological morbidity is positively related to IA. First, depression has been widely identified as a key risk factor for IA. There are several accounts of how depression is linked to IA. According to the Cognitive and Behavioral Model (CBM) of pathological Internet use, maladaptive cognitive and behavioral patterns contribute to the development of IA (Davis, 2001). Individuals with depressive symptoms may be more prone to developing IA due to cognitive distortions and biases closely related to depression, such as a ruminative cognitive style and negative self-appraisal. Besides, according to the Mood Enhancement Hypothesis (Liang et al., 2016), individuals with depression tend to engage more in leisure activities such as surfing the Internet and playing online games to deal with stress. Finally, the “Interaction of the Person-Affective-Cognition-Execution” (I-PACE) Model (Brand et al., 2016) posits that depression can impair attention, executive functioning, and decision-making, which would predispose individuals to IA.

In line with the theoretical propositions, empirical studies have shown a significant positive relationship between depressive symptoms and IA (Ali et al., 2021; Rachubińska et al., 2021; Zhao et al., 2022). Such a relationship also occurs among university students during COVID-19 (Fawaz and Samaha, 2021; Gavurova et al., 2022; Mengistu et al., 2023). With particular reference to Hong Kong, findings showed that depressive symptoms were positively related to IA among university students during the pandemic (Shek et al., 2023a, 2023b).

Another risk factor of IA during the pandemic is loss of hope (i.e., hopelessness). Hope is an important protective factor among university/college students under COVID-19 because it is an internal force that helps people to cope when facing pain and stressful events (Öztunç et al., 2013; Madani et al., 2018). Having hope is fundamental to life satisfaction (Shek, 2010) and acts as a protection from developing IA since it consists of positive thoughts regarding our future which motivates and develops our adaptive skills when encountering any stressful situations (Shek et al., 2023a). In contrast, hopelessness consists of a negative perception of the future which would intensify an individual’s addictive behaviors such as IA, particularly during the pandemic which is a chronic stressor. Regarding the relationship between hopelessness and IA, there are three observations. First, there are few studies in this area particularly during the pandemic. Second, as hopelessness is a form of psychological morbidity, we can argue that hopelessness would be positively associated with IA during the pandemic. Third, there are conflicting findings on the relationship between hopelessness and IA. While studies showed that hopelessness was an antecedent of IA (Chen et al., 2021), Yu and Shek (2018) found that IA was a predictor of hopelessness, not a consequence of IA over time.

Suicidal behavior is also a form of psychological morbidity closely related to IA. Existing studies have shown a significant correlation between IA and suicidal ideation where IA is regarded as a potent risk factor for suicidal behavior (Steinbüchel et al., 2018; Arrivillaga et al., 2020). Several studies indicated that students with IA experience more culturally defined failures in their real lives (Yee, 2006), such as lower academic achievements, which make them more prone to engaging in suicidal behavior (Platt et al., 2013). Besides, students with IA are more likely to access harmful information and develop suicidal thoughts (Baker and Fortune, 2008; Biddle et al., 2008). However, limited studies have focused on the relationship between IA and suicidal behavior among Hong Kong university students. In addition, fewer studies have examined the predictive role of suicidal ideation in IA, where those who are suicidal may turn to IA to cope with the pain.

### 1.3. Interaction effects of socio-demographic factors and psychological morbidity

Regarding gender differences in IA, the basic picture is that men have significantly higher rates of IA than women (Anand et al., 2018; Onukwuli et al., 2023), although there are minor exceptions (Yang et al., 2021). Obviously, it is important to ask whether gender would interact with other factors to shape IA. Theoretically, the I-PACE model (Brand et al., 2019) highlights the moderating role of gender in the development of IA. This model contends that the emergence of addictive behaviors results from the interaction of personal core characteristics (e.g., gender) and internal and/or external stressors (e.g., the COVID-19 pandemic). Therefore, gender may interact with psychological morbidity as an internal stressor to trigger IA.

Empirically, some studies showed that gender moderated the relationship between psychological issues and IA, with a stronger relationship between these constructs found in males than females. According to Liang et al. (2016), there are gender variations in the causal link between depression and IA among Chinese adolescent students (*N* = 1,715) where depression did only significantly predict IA in male adolescents but did not in female adolescents. Likewise, Li et al. (2021) revealed that the interaction of gender and well-being significantly predicted smartphone addiction in adolescents. Compared with boys, girls’ smartphone addiction scores decreased more when there was an improvement in well-being. However, some studies did not show a significant interaction effect of gender and depressive symptoms on IA (Masaeli and Farhadi, 2021; Son et al., 2021; Liu and Lu, 2022). Hence, there is a need to further explore the interaction effect of gender and psychological morbidity (such as depression, hopelessness, and suicidal behavior) on IA.

### 1.4. Personal infection of COVID-19 as a moderator of the relationship between psychological morbidity and IA

A review of the literature (Oka et al., 2021; Sultana et al., 2021) shows that there are limited studies on the moderating role of personal infection of COVID-19 on the relationship between psychological morbidity and IA. Logically speaking, it can be reasoned that infection of COVID-19 would intensify the relationship between psychological morbidity and IA because of the stressful nature of COVID-19. Based on the Compensation Theory of IA, individuals may use the Internet excessively to reduce negative emotions caused by negative life events in their lives (Kardefelt-Winther, 2014). Wang et al. (2015) applied the theory to examine the moderating effect of stress on the association between escapism and problematic smartphone use in university students. There are also studies on the significant association between adolescents’ stress and problematic behavior (Liu et al., 2005; Xing et al., 2010). Studies on the mental health of COVID-19-infected individuals highlighted that the infection was a significant factor contributing to mental distress (Mazza et al., 2020; Ochnik et al., 2021; Sultana et al., 2021). These findings indicate that infection with COVID-19 would be a significant stressor for university students. As both the I-PACE model and the Compensation Theory of IA emphasize the influence of the interaction between individuals’ mental status (internal stressors) and stressful life events (external stressors) on the development of IA, infection with COVID-19 may interact with psychological morbidity to predict IA.

### 1.5. The present study

To address the research gaps identified in the above literature review, this study aimed to investigate the predictive role of socio-demographic factors and psychological morbidity in IA, and the moderating effects of gender and personal infection of COVID-19 on the relationship between psychological morbidity and IA. we framed several research questions in this study as follows:

Research Question 1: What are the socio-demographic correlates of IA in university students under COVID-19? Particularly, with reference to financial difficulty, we hypothesized a positive relationship between financial difficulty and IA (Hypothesis 1).
Research Question 2: What is the relationship between psychological morbidity and IA? We hypothesized a positive relationship between psychological morbidity and IA (Hypothesis 2).
Research Question 3: What is the interactive effect between gender and psychological morbidity on IA? We hypothesized that gender would moderate the association between psychological morbidity and IA (Hypothesis 3).
Research Question 4: What is the interactive effect between infection with COVID-19 and psychological morbidity on IA? We proposed that infection with COVID-19 would intensify the association between psychological morbidity and IA (Hypothesis 4).

## 2. Methods

### 2.1. Participants and procedure

We conducted an online survey during the ending phase of the fifth wave of the pandemic in Hong Kong (i.e., from late 2022 to early 2023) to collect data from first-year undergraduates at a university. During this period, the pandemic in Hong Kong was in its early stages of recovery (Bala, 2023). The Government of the Hong Kong Special Administrative Region (2022) had adjusted its local epidemic prevention policy to allow citizens to gradually resume normal life. It has also announced policies to promote economic recovery after the pandemic and incentive measures to support businesses and residents. Along with the mitigation of the pandemic, the university resumed face-to-face teaching and learning in September 2022, requiring undergraduate students to attend all courses on campus.

Quota sampling was employed, with faculty as the stratifying factor. For participant recruitment, part-time student helpers were recruited from undergraduate students in different faculties to invite first-year students to participate in the study through different means such as email, personal contacts, and peer recommendation. Initially, 1,043 first-year students completed the online survey questionnaire. Among these, 23 students did not pass the “attention checking” test designed to improve the data validity of the online survey (Aust et al., 2013). This test gauged respondent conscientiousness by instructing them to select a specified option (e.g., “This is an attention check, please choose ‘strongly agree’”). Following this, we had a total of 1,020 valid questionnaires. The questionnaire was conducted in English, which is the primary medium of instruction at this institution. Prior to their participation, students were informed about the study purpose, confidentiality, anonymity, and the voluntary nature of their participation. Students were given the option to withdraw their participation at any time during or prior to the completion of the questionnaire. We offered a supermarket coupon (HK$100 ≈ US$12.74) to each participant who completed the survey. The institutional review board of the University granted ethical approval for this study.

### 2.2. Measure

#### 2.2.1. Internet addiction (IAT-10)

We employed the Chinese version of “Young’s 10-item Internet Addiction Test (IAT-10),” which was developed based on the original English version (Young, 1998), to assess the presence of IA among student participants. The IAT-10 consists of ten items that measure addictive symptoms related to Internet use, such as preoccupation with the Internet, loss of control over Internet use, and negative consequences of Internet use. The students needed to indicate whether they had experienced the ten listed symptoms in the past year using a binary reporting scale (“Yes” or “No”). A sample item is “*Do you feel a need to spend more and more time online to achieve satisfaction?*” This scale has demonstrated adequate psychometric properties in previous research (Shek et al., 2008) and has been widely used in the population of adolescents (Fioravanti et al., 2013) and college students (Samaha et al., 2018; Shek et al., 2023a). The scale also showed good internal consistency with Cronbach’s alpha = 0.83 and mean inter-item correlation = 0.32 (see Table 1) in the present investigation.

**Table 1:** Descriptive analysis of variables.

	Mean	Std. Deviation	Cronbach’s *α*	Mean inter-item correlation
Internet addiction (IA)	0.360	0.291	0.826	0.321
Depression (CESD-R)	0.763	0.642	0.947	0.477
Suicidal behavior (SB)	0.090	0.208	0.597	0.409
Hopelessness (HL)	3.125	0.959	0.867	0.565

#### 2.2.2. Depression (CESD-R)

The “Centre for Epidemiologic Studies Depression Scale Revised (CESD-R)” was utilized as a self-report measure to assess depression. CESD-R is a 20-item measure originally developed by Radloff (1977) and subsequently revised by Eaton et al. (2004), which evaluates the symptoms of major depressive disorder as specified in the fifth edition of the “Diagnostic and Statistical Manual of Mental Disorders” (DSM-V) criteria. Respondents were instructed to indicate how frequently they experienced each symptom in the past week on a scale of five points, ranging from “0 = Not at all or less than 1 day” to “4 = nearly every day for the last 2 weeks.” A sample item is “*I lost interest in my usual activities.*” The composite score was the sum of all item scores. The CESD-R has revealed desirable psychometric properties in various cultural contexts, including the Chinese context (e.g., Ip et al., 2016; Dou et al., 2021; Zhu et al., 2021). In this study, the internal consistency of the CESD-R was high, with Cronbach’s alpha being 0.95 and mean inter-item correlation being 0.48 (see Table 1).

#### 2.2.3. Suicidal behavior

The Suicidal Behavior Scale (SB) was used to measure suicidal behavior (Shek and Yu, 2012), which consists of three items, namely “suicidal thoughts,” “suicidal plans,” and “suicidal attempts.” Respondents were required to indicate their experience of the aforementioned items during the past year by answering on a binary scale (“Yes” or “No”). A sample item is “*In the past year, have you ever had suicidal thoughts?*” The reliability of SB has been established in previous research (Shek and Yu, 2012; Law and Shek, 2013). In the present study, Cronbach’s alpha for the three items was 0.60 (see Table 1). The relatively lower alpha value may be due to the small number of items (3 items) in this measure (Streiner, 2003). However, different research reported different acceptable values of Cronbach’s α for a scale, ranging from 0.61 to 0.95 (Bland and Altman, 1997). So, the Cronbach’s α value of 0.60 is close to the acceptable value and the scale could be regarded as internally consistent. In addition, three items were positively correlated with each other with the mean inter-item correlation = 0.41, slightly greater than the ideal interval of 0.2–0.4, suggesting that while the items are reasonably homogeneous and measure the same construct, they do contain sufficiently unique variance that they cannot be isomorphic to each other (Piedmont, 2014).

#### 2.2.4. Hopelessness (C-HOPE)

The level of hopelessness in university students was assessed by the modified Chinese version of the “Hopelessness Scale” (C-HOPE; Shek, 1993) which showed good psychometric properties (Kwok and Shek, 2010; Shek and Li, 2016; Zhou et al., 2020). The respondents were asked to indicate to what degree they agree with each item using a six-point rating scale, ranging from “1 (strongly disagree)” to “6 (strongly agree).” A sample item is “*I could foresee that my future is miserable*.” Reliability analysis showed that the scale is internally consistent (Cronbach’s alpha = 0.87 and mean inter-item correlation = 0.57, see Table 1) in the present investigation.

#### 2.2.5. Socio-demographic variables

We collected socio-demographic data from the respondents, including age (continuous values), gender (male vs. female), student status (local vs. international), living status (living with family and roommates vs. living alone), “Comprehensive Social Security Assistance (CSSA) Scheme” status (receive vs. not receive), family financial difficulty (yes vs. no), personal financial difficulty (yes vs. no), family member(s) unemployment (yes vs. no), personal infection with COVID-19 (yes vs. no), and family infection with COVID-19 (yes vs. no).

### 2.3. Data analysis strategy

We utilized SPSS 26.0 for data analysis. Descriptive analyses were first performed which included the socio-demographic characteristics of the sample and descriptive statistics of all major variables (mean scores, standard deviation, Cronbach’s alpha and mean inter-item correlation). We also conducted correlation analyses to examine the inter-correlations among all related variables. To examine the predictive role of socio-demographic variables and psychological morbidity in IA and the interactive effect of gender and personal infection with COVID-19 on psychological morbidity in IA, hierarchical regression analyses were conducted with socio-demographic variables included in the model in the first step, psychological morbidity variables included in the second step and interaction between gender, personal infection with COVID-19 and psychological morbidity entered in the model in the third step. Finally, to further explore the significant interaction effects, simple slope analyses were performed.

## 3. Results

Table 2 shows the socio-demographic characteristics of the sample participants. The mean age of the participants was 18.7 years (SD = 1.46), and around half of the participants were female (50.7%). The majority of the participants were local students (84.1%), and most were living with their family (84.0%). A small proportion of the students were living with their roommates (14.6%) or living alone (1.4%). Besides, most of the students were not recipients of CSSA (88.4%), which is a government financial subsidy for low-income Hong Kong residents. Regarding the financial difficulty during the COVID-19 pandemic, 18.0% and 24.3% of the students reported experiencing family and personal financial difficulty, respectively. A small proportion of students had unemployed family members during the pandemic (14.2%). Around half of the students were infected with COVID-19 (46.6%) or had infected family member(s) (58.8%).

**Table 2:** Socio-demographic characteristics of the sample.

	*N*	%
Gender of the participant		
Male	451	44.2%
Female	517	50.7%
Local or international student		
Local student	858	84.1%
International student	162	15.9%
Living status during COVID-19		
Live with family	857	84.0%
Live with roommates	149	14.6%
Live alone	14	1.4%
Recipient of CSSA		
No	902	88.4%
Yes	40	3.9%
Family financial difficulty		
No	684	67.1%
Yes	184	18.0%
Personal financial difficulty		
No	682	66.9%
Yes	248	24.3%
Family member(s) unemployed during COVID-19		
No	810	79.4%
Yes	145	14.2%
Personal infection of virus		
No	508	49.8%
Yes	475	46.6%
Family member(s) infection of virus		
No	369	36.2%
Yes	600	58.8%
	Mean	SD
Age	18.7	1.46

The correlations amongst all the variables were shown in Table 3. Results showed that the four major variables (IA, CESD-R, HL and SB) were positively correlated with each other (*rs* = 0.18–0.47, *ps* < 0.01). In addition, the four major variables were correlated with some of the socio-demographic variables. Particularly, the four major variables were all positively correlated with family and personal financial difficulty (*rs* = 0.09–0.19, *ps* < 0.05 and *ps* < 0.01). IA and CESD-R were also positively correlated with family member(s) being unemployed (*rs* = 0.09–0.10, *ps* < 0.05).

**Table 3:** Correlation analysis.

	1	2	3	4	5	6	7	8	9	10	11	12	13
IA	-												
CESD-R	0.381**	-											
HL	0.247**	0.471**	-										
SB	0.314**	0.321**	0.182**	-									
Age	−0.020	−0.001	−0.103**	−0.007	-								
Gender	0.012	−0.010	−0.055	0.016	−0.072*	-							
Local vs. International	0.046	−0.027	−0.231**	0.063	0.022	0.004	-						
Living status	0.001	0.004	−0.117**	0.038	0.047	0.098**	0.471**	-					
CSSA	−0.014	0.021	0.082*	0.087*	−0.016	0.016	0.169**	0.065	-				
Family financial difficulty	0.113**	0.175**	0.087*	0.184**	0.047	0.033	−0.009	0.075*	0.154**	-			
Personal financial difficulty	0.135**	0.161**	0.112**	0.185**	0.110**	0.000	−0.021	0.119**	0.058	0.636**	-		
Family member unemployed	0.095**	0.090*	0.043	0.062	0.096**	−0.018	−0.062	0.038	0.079*	0.383**	0.332**	-	
Personal infection of virus	0.002	0.007	0.008	−0.002	−0.021	−0.012	−0.086*	−0.052	0.035	0.099**	0.118**	0.114**	-
Family infection of virus	−0.032	−0.056	0.017	0.01	0.072*	−0.034	−0.175**	−0.113**	−0.065	0.007	0.095**	0.031	0.464**

^*^p < 0.05, ^**^p < 0.01; IA = Internet addiction, CESD-R = Depressive symptoms, HL = Hopelessness, SB = Suicidal behavior.

Hierarchical multiple regression analyses were performed to examine the predictive effect of the socio-demographic variables, the three psychological morbidity variables (CESD-R, SB and HL), and the interaction between socio-demographic variables (i.e., gender and personal infection of COVID-19) and psychological morbidity on IA (see Table 4). In the first step, the socio-demographic variables were put in the model as independent variables. Among all the socio-demographic variables, only personal financial difficulty significantly predicted IA (*β* = 0.13, *p* < 0.01). Therefore, Hypothesis 1 was supported. In the second step, all three variables of psychological morbidity (CESD-R, HL, SB) were put in the model as predictors. The three psychological morbidity variables all significantly and positively predicted IA (*β* = 0.09–0.26, *p* < 0.001 and *p* < 0.05), which supported Hypothesis 2. In the third step, the interaction between the two demographic variables (gender and the personal infection of COVID-19) and the three psychological morbidity variables (CESD-R, SB and HL) were analyzed, respectively. For the moderating role of gender, gender negatively moderated the association between CESD-R and IA (*β* = −0.30, *p* < 0.05), with the association being stronger in male students than in female students. In contrast, gender positively moderated the association between HL and IA (*β* = 0.31, *p* = 0.01), with the association being stronger in female students than in male students. Besides, there was no moderating effect of gender on the association between SB and IA. Therefore, Hypothesis 3 was partially supported. For the moderating effect of personal infection of COVID-19, it positively moderated the association between SB and IA (*β* = 0.13, *p* < 0.01) but did not moderate the association between the two other psychological morbidities and IA. Hypothesis 4 was partially supported.

**Table 4:** Predictive effects and interactions.

Predictor	*B*	*SE*	*β*	*F* change	*R*^2^ change
*Step 1* demographic variables				2.26^*^	0.03
Age	−0.05	0.07	−0.03		
Gender	0.05	0.21	0.01		
Student status	0.49	0.35	0.05		
Living status	−2.18	1.31	−0.06		
Recipient of CSSA	−0.52	0.52	−0.04		
Family financial difficulty	−0.02	0.36	−0.003		
Personal financial difficulty	0.86^**^	0.33	0.13		
Family member(s) unemployed	0.55	0.33	0.07		
Personal infection of virus	0.00	0.24	0.00		
Family infection of virus	−0.17	0.25	−0.03		
*Step 2* independent variables				53.05^***^	0.18
Depressive symptoms (CESD)	0.06^***^	0.01	0.26		
Suicidal behavior (SB)	1.00^***^	0.16	0.22		
Hopelessness (HL)	0.27^*^	0.12	0.09		
*Step 3a* interaction with gender^a^				3.05^*^	0.01
CESD×gender	−0.54^*^	0.22	−0.30		
SB × gender	−0.04	0.20	−0.02		
HL × gender	0.57^b^	0.22	0.31		
*Step 3b* interaction with COVIDP^a^				2.53^ms^	0.01
CESD×COVIDP	−0.15	0.22	−0.04		
SB × COVIDP	0.55^**^	0.20	0.13		
HL × COVIDP	−0.09	0.22	−0.02		

a interactions between each demographic variable and all psychological morbidity were examined.

ms marginal significance (*p* = 0.06).

b *p* = 0.01.

^*^p < 0.05, ^**^p < 0.01, ^***^p < 0.001. COVIDP = Personal Infection with COVID-19.

**Figure 1:**
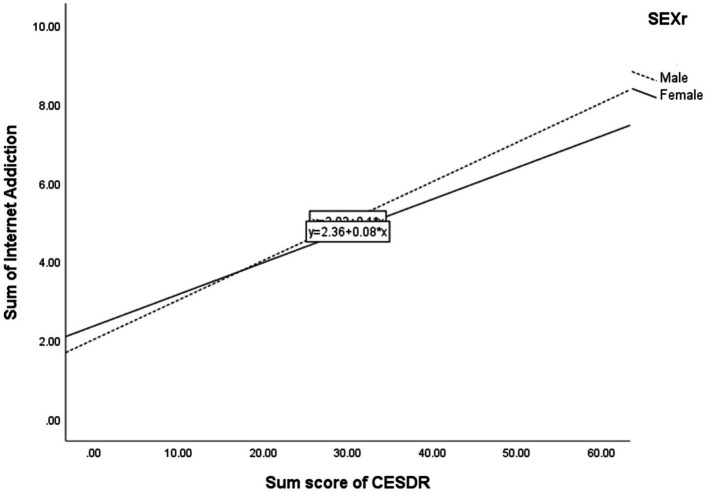
Interaction between symptoms of major depressive disorder (CESDR) and gender.

**Figure 2:**
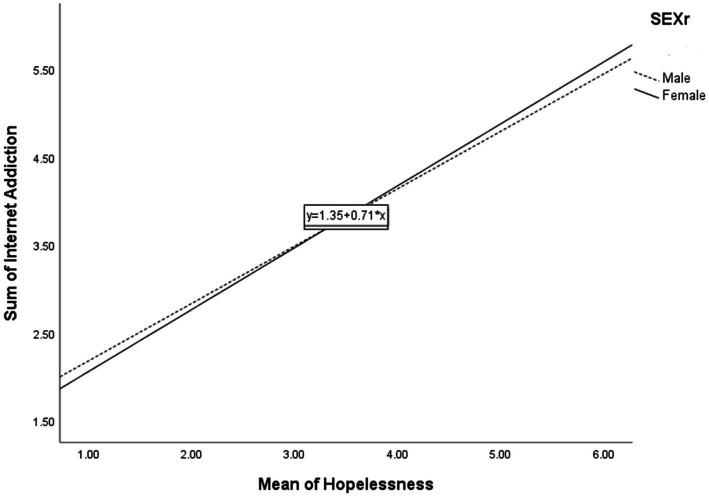
Interaction between hopelessness and gender.

**Figure 3:**
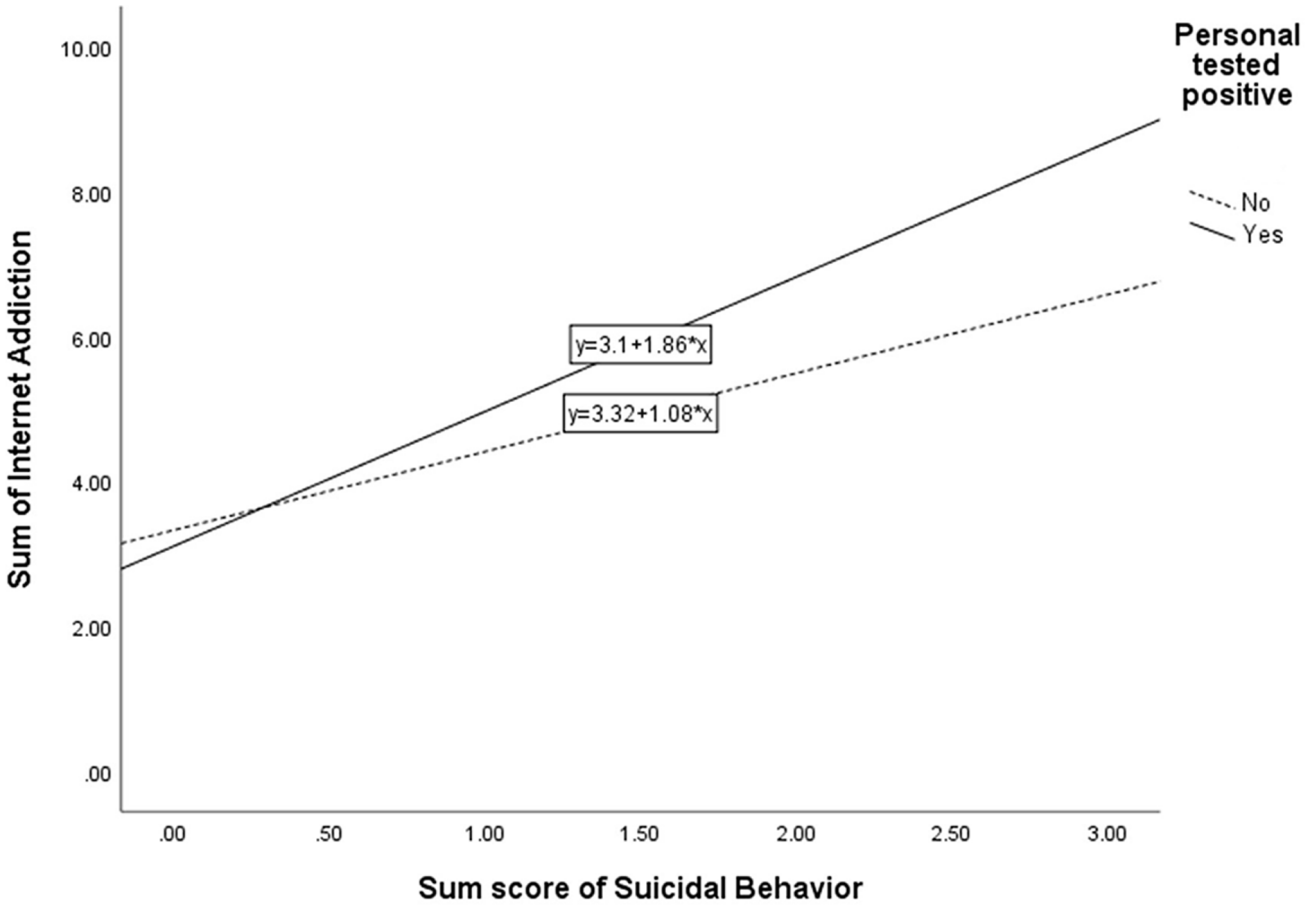
Interaction between suicidal behavior and personal infection of virus.

Table 5 shows the simple slope analyses on the above significant interaction effects. First, for the regression of IA on CESD-R, male students (*β* = 0.45, *p* < 0.001) had a higher regression coefficient than did female students (*β* = 0.35, *p* < 0.001). Figure 1 illustrates the moderating effect graphically. Second, for the regression of IA on HL, female students (*β* = 0.23, *p* < 0.001) had a higher regression coefficient than did male students (*β* = 0.22, *p* < 0.001). Figure 2 graphically illustrates this moderating effect. Third, for the regression of IA on SB, simple slope analyses showed that the regression coefficient of the relationship was higher among students who were previously infected with the virus (*β* = 0.39, *p* < 0.001) than students who were not (*β* = 0.23, *p* < 0.001). Figure 3 graphically illustrates this moderating effect.

**Table 5:** Simple slope analyses.

Path	Moderator	Category	*B*	*SE*	*β*
*CESD to IA*					
	Gender	Female	0.08^***^	0.01	0.35
		Male	0.10^***^	0.01	0.45
*HL to IA*					
	Gender	Female	0.71^***^	0.13	0.23
		Male	0.65^***^	0.14	0.22
*SB to IA*					
	COVIDP	Yes	1.49^***^	0.20	0.39
		No	1.09^***^	0.21	0.23

^***^p < 0.001.

## 4. Discussion

This study examined the association between socio-demographic factors, psychological morbidity and IA in university students in Hong Kong during the pandemic. The study is significant in several aspects. First, there are limited studies examining socio-demographic factors and psychological morbidity as predictors of IA in university students during the COVID-19 pandemic, particularly in the context of Hong Kong which is very densely populated with both Chinese and Western cultural influences. Second, the study promotes our understanding of the role of socio-demographic factors in IA during the pandemic, particularly the relevance of personal infection and financial difficulty. Third, consistent with existing studies, the present study reiterated the crucial role of psychological morbidity as a risk factor for IA. Finally, this study contributes to our understanding of the interaction effects of some socio-demographic factors (i.e., gender and infection of COVID-19) and some psychological morbidity conditions during the COVID-19 pandemic on IA. The present findings are important for understanding individual differences in IA because there are very few studies looking at the interaction of different ecological factors on IA.

Existing literature highlights individual differences in IA. Particularly, the literature indicates the prevalence and severity of IA differ in individuals with different socio-demographic characteristics (e.g., Chi et al., 2020; Gavurova et al., 2022) and psychological morbidity (e.g., Arrivillaga et al., 2020; Fawaz and Samaha, 2021). While research studies suggest that university students are vulnerable to IA, students with some attributes are more vulnerable than others if risk factors are operating. During the pandemic, some individual factors such as financial difficulty and infection with the virus may have a unique impact on IA. Furthermore, students’ psychological morbidity would be intensified during the pandemic, which may also promote IA.

### 4.1. Socio-demographic predictors of IA

The present study showed that personal financial difficulty during the pandemic significantly predicted IA after controlling for the effects of other socio-demographic factors. This highlights the important risk role that personal financial difficulty plays in IA during the pandemic. While the general literature suggests an association between economic status and IA, it mainly focuses on adolescents and the findings are inconclusive (Ak et al., 2013; Wu et al., 2016; Lai and Kwan, 2017). A recent study on 1,648 university students in Hong Kong showed that personal financial difficulty rather than family financial difficulty predicted IA during the pandemic (Shek et al., 2023a). The finding of the present study aligns with this finding and the conjecture that economic problems are closely linked with adolescent developmental outcomes (Leung and Shek, 2011). Compared to adolescents who mainly rely on their families for economic support, many university students would find part-time work to financially support themselves or even to shoulder family financial burdens (Richardson et al., 2009; Shek et al., 2022a). Unfortunately, during the pandemic, university students face difficulties in finding part-time work due to the economic downturn (Adams-Prassl et al., 2020), which may lead to increased stress and subsequent problem behaviors such as IA.

Regarding gender, results of the present study showed that gender did not predict IA. While some studies found that male students showed higher IA than female students (Gavurova et al., 2022; Kożybka et al., 2023), some studies showed the reverse pattern (e.g., Shehata and Abdeldaim, 2021; Mengistu et al., 2023). Some studies also showed no significant gender effect on IA in university students (e.g., Truzoli et al., 2016; Shek et al., 2023a). The observation of no gender difference may be due to the wide accessibility of the Internet, the diversification of functions of the Internet, and the long time spent on the Internet by both males and females during the pandemic (Seyrek et al., 2017; Talwar et al., 2019). In other words, while gender differences may exist under “normal” circumstances before COVID-19, gender differences may disappear under “non-normal” conditions such as the pandemic because both males and females spend more time on the Internet.

### 4.2. Psychological morbidity predictors of IA

The present study showed that psychological morbidity including depression, suicidal behavior and hopelessness positively predicted IA. This is in line with the extant literature. For depression, a body of research showed a positive relationship between depression and IA including studies conducted during the pandemic (Ali et al., 2021; Chen et al., 2021; Fawaz and Samaha, 2021). Findings of this study provide further evidence to the literature. In addition, findings of this study advance our understanding of the relationship between some specific psychological morbidity such as suicidal behavior and IA. While previous studies mainly suggest IA as a predictor of suicidal behavior (Arrivillaga et al., 2020), findings of the present study indicate that suicidal behavior may be a potential risk factor for IA. This conjecture is consistent with the Compensation Theory that IA may be a coping response to stress (e.g., stress created by suicidal ideation). Besides, individuals with suicidal intentions may use IA to distract themselves to delay the suicidal act. This novel insight merits further investigation in longitudinal studies.

Regarding hopelessness, a few studies examined the risk role of hopelessness in IA. For example, a study found that hopelessness predicted Internet abuse in college students (Velezmoro et al., 2010). Another study showed that hopelessness mediated the relationship between attention deficit/hyperactivity disorder and Internet gaming disorder (Chen et al., 2021). In a recent study on Hong Kong university students, hopelessness was positively correlated with IA (Shek et al., 2023a). Results of the present study echo these findings and suggest that hopelessness may be a risk factor for IA. Overall, the findings on the predictive effects of these three domains of psychological morbidities on IA provide support to the Compensation Theory and the I-PACE model of IA which highlight that psychological morbidity or mental illness is a major stressor leading to IA (Brand et al., 2016).

### 4.3. Gender as a moderator in the predictive relationship between depression and IA

While gender did not have a main effect on IA, it moderated the association between psychological morbidity (depression and hopelessness) and IA in the present study. For depression, results of this study showed that compared to female students, depressive symptoms had a stronger impact on IA in male students. This contributes to the existing yet inconclusive literature. While some studies showed that depressed male students were more likely to have IA or Internet gaming disorder than depressed female students (Liang et al., 2016; Gan et al., 2022), other studies revealed that depression predicted smartphone addiction only in female students (Koh and Kim, 2017) or gender did not moderate the association between early social media disorder (a specific type of IA) and depressive symptoms (Bos, 2018). Conceptually, male and female students may have different motivations for Internet use due to their different gender identities formed under diverse societal expectations (Chen et al., 2017), which may influence the relationship between their mental health problems and behavioral outcomes (Gan et al., 2022). For male students, societal and cultural expectations shape their gender roles to emphasize social status, accomplishments, and power (Chen et al., 2017). Failing to meet these expectations may result in feelings of depression. Through the Internet, especially online games, males could gain a “false sense of power and achievement” which helps them to regulate or reduce the negative feelings associated with depression (Kwon, 2007, p. 231). Consequently, they may be more likely to use the Internet when feeling depressed.

### 4.4. Gender as a moderator in the predictive relationship between hopelessness and IA

Different from the moderating effect of gender on the association between depression and IA, we observed a novel finding that the link between hopelessness and IA was stronger in girls than did boys. It indicates that the moderating function of gender might be different for the linkage between different forms of psychological morbidities and IA. As suggested by the existing literature, while hopelessness is associated with depression (Polanco-Roman and Miranda, 2013; Horwitz et al., 2017), they are distinctive constructs (DeLisle and Holden, 2009) and hopelessness may only be associated with certain types of depression (Alloy and Clements, 1998; Joiner et al., 2001). Scholars also pointed out that there were hopeless but non-depressed individuals (Young et al., 1996) and depressed but non-hopeless individuals (Greene, 1989). As such, the underlying mechanisms might be different for the moderating function of gender in the association between these constructs and IA. For example, one study found that hopelessness rather than depression was a significant predictor of interpersonal stress (Joiner et al., 2005) or was an outcome of negative interpersonal events (Abela and Seligman, 2000). As girls tend to use social media or social networks for social connection and boys tend to play video games in their Internet behaviors (Dufour et al., 2016; Leonhardt and Overå, 2021), hopeless female students with interpersonal problems may resort to social media to compensate for their interpersonal handicaps, thus leading to higher IA. The finding is also consistent with the meta-analytic review that the moderating role of gender in the relationship between different psychological morbidities and IA would be different (Cai et al., 2023).

### 4.5. Personal infection as a moderator in the predictive relationship between suicidal behavior and IA

The present study showed that personal infection moderated the association between suicidal behavior and IA. While the finding is novel, some existing studies may imply the possible moderating role of personal infection in the suicidality-IA link. For example, research showed that COVID-19 infection was an important risk factor for suicidal behavior during the pandemic (Raj et al., 2020; Shi et al., 2021). COVID-19 patients may have neurological problems or disorders such as “ischemic stroke and headaches” and have increased fear which would increase their risk for suicidal behavior (Raj et al., 2020, p. 4). Based on this background, COVID-19 infection may enhance the existing level of suicidal behavior which then strengthens its association with IA. In fact, both the I-PACE model (Elhai et al., 2020) and the Compensation Theory of IA (Kardefelt-Winther, 2014) highlight the association between external stressors (COVID-19 infection in this case) and internal stressors (suicidal behavior) in the development of IA. According to these views, the infection of COVID-19 may interact with suicidal behavior to increase the related physical symptoms and negative mood which then leads to higher IA behavior.

### 4.6. Implications and limitations of the study

In addition to the theoretical implications mentioned above, the study also has practical implications. Firstly, understanding the risk role of personal financial difficulty and psychological morbidity would help university mental health practitioners and policymakers in identifying student groups who are more vulnerable to IA during the pandemic for targeted service provision, prevention and intervention. Secondly, the risk role of psychological morbidity in IA suggests that the treatment of IA should involve assessment and treatment of psychological morbidity. Thirdly, the moderating roles of gender and personal infection of COVID-19 in the relationship between psychological morbidity and IA indicate that treatment of IA that involves addressing psychological morbidity should account for the different moderating roles of gender in the relationship between specific types of psychological morbidity and IA. Furthermore, the treatment of IA that involves addressing psychological morbidity should pay special attention to the student group who have personal experience with infection of COVID-19.

Despite the novel nature of the study, several limitations of this study should be noted. First, a cross-sectional design was employed in this study which cannot infer any causal relationship. Hence, longitudinal research should be conducted in future to examine the related issues. Second, due to difficulties in data collection under COVID-19, the study adopted the method of non-probability quota sampling which was commonly adopted in other studies (Chaabna et al., 2022). Third, although many studies under COVID-19 were also based on students from a single university (e.g., Ismail et al., 2021; Shehata and Abdeldaim, 2021), future research should be based on students from different universities. Despite these limitations, the study contributes significantly to the area of individual differences in IA particularly the role of socio-demographic factors and psychological morbidity, as well as their interaction, in IA in university students during the pandemic.

## Data availability statement

The raw data supporting the conclusions of this article will be made available by the authors, without undue reservation.

## Ethics statement

The studies involving humans were approved by the Institutional Review Board (or its Delegate) at the Hong Kong Polytechnic University. The studies were conducted in accordance with the local legislation and institutional requirements. The participants provided their written informed consent to participate in this study.

## Funding

The preparation of this paper is financially supported by the matching funds from a project funded by Chow Tai Fook Charity Foundation and Keswick Foundation (PolyU Matching: 52U9; Research Grants Council: ZECL) as well as the Li and Fung Endowed Professorship.

## Author contributions

DS obtained the research grant, conceived the research, contributed to all stages of the research work, and critically revised all versions of the manuscript. WC conducted data analyses, drafted parts of the manuscript, and revised and checked the manuscript. DD contributed to the research design, and revised, checked and proofread the manuscript. LT drafted parts of the manuscript and checked the manuscript. TW drafted parts of the manuscript and checked the manuscript. KZ drafted parts of the manuscript. All authors contributed to the article and approved the submitted version.

## Conflict of interest

The authors declare that the research was conducted in the absence of any commercial or financial relationships that could be construed as a potential conflict of interest.

## Publisher’s note

All claims expressed in this article are solely those of the authors and do not necessarily represent those of their affiliated organizations, or those of the publisher, the editors and the reviewers. Any product that may be evaluated in this article, or claim that may be made by its manufacturer, is not guaranteed or endorsed by the publisher.
